# Ventilation Improvements Among K–12 Public School Districts — United States, August–December 2022

**DOI:** 10.15585/mmwr.mm7214a4

**Published:** 2023-04-07

**Authors:** Miguella Mark-Carew, Gloria Kang, Sanjana Pampati, Kenneth R. Mead, Stephen B. Martin, Lisa C. Barrios

**Affiliations:** ^1^Global Government Solutions, San Antonio, Texas; ^2^Division of Preparedness and Emerging Infections, National Center for Zoonotic and Emerging Infectious Diseases, CDC; ^3^Division of Adolescent and School Health, National Center for HIV, Viral Hepatitis, STD, and TB Prevention, CDC; ^4^Division of Field Studies and Engineering, National Institute for Occupational Safety and Health, CDC; ^5^Respiratory Health Division, National Institute for Occupational Safety and Health, CDC.

Improving ventilation has been one of several COVID-19 prevention strategies implemented by kindergarten through grade 12 (K–12) schools to stay open for safe in-person learning. Because transmission of SARS-CoV-2 occurs through inhalation of infectious viral particles, it is important to reduce the concentration of and exposure time to infectious aerosols (*1*–*3*). CDC examined reported ventilation improvement strategies among U.S. K–12 public school districts using telephone survey data collected during August–December 2022. Maintaining continuous airflow through school buildings during active hours was the most frequently reported strategy by school districts (50.7%); 33.9% of school districts reported replacement or upgrade of heating, ventilation, and air conditioning (HVAC) systems; 28.0% reported installation or use of in-room air cleaners with high-efficiency particulate air (HEPA) filters; and 8.2% reported installation of ultraviolet (UV) germicidal irradiation (UVGI) devices, which use UV light to kill airborne pathogens, including bacteria and viruses. School districts in National Center for Education Statistics (NCES) city locales, the West U.S. Census Bureau region, and those designated by U.S. Census Bureau Small Area Income Poverty Estimates (SAIPE) as high-poverty districts reported the highest percentages of HVAC system upgrades and HEPA-filtered in-room air cleaner use, although 28%–60% of all responses were unknown or missing. Federal funding remains available to school districts to support ventilation improvements. Public health departments can encourage K–12 school officials to use available funding to improve ventilation and help reduce transmission of respiratory diseases in K–12 settings.

MCH Strategic Data (MCH), a private company offering educational, health care, and government data and technology solutions, repeatedly surveyed school district office staff members by telephone during August 8–December 29, 2022, regarding implementation of COVID-19 prevention policies and strategies, including ventilation improvements. Ventilation strategies examined included replacing or upgrading HVAC systems; using in-room air cleaners with HEPA filters; installing UVGI in high-risk areas; and maintaining continuous movement of air supply or airflow through school buildings during active hours, including opening windows and doors, using fans, or adjusting thermostats or central controls.* School districts were asked, “Since the start of the COVID-19 pandemic, which of the following steps to increase ventilation or filter/clean air apply to most schools (at least 50%) in your district?”

MCH attempted to contact 15,871 U.S. K–12 public and public charter school districts^†^ (estimated student enrollment = 52,696,479) at a cadence based on district enrollment size, whereby larger school districts (those with 2,500 or more students) were surveyed weekly, and districts with less than 2,500 students were surveyed biweekly. The most recent response from school districts was included for analysis. Individual school-level enrollment was summed for each district.

Although 1,358 independent charter school districts were surveyed, they were excluded from this analysis because SAIPE school district data were not available for these districts. Descriptive analyses summarizing responses to ventilation questions are presented for the overall sample and stratified by U.S. Census Bureau region,^§^ NCES locale,^¶^ and poverty level. Poverty levels were based on the 2021 SAIPE for school-aged children living in poverty** as the percentage of children and adolescents aged 5–17 years experiencing poverty within a school district, grouped into tertiles. Responses were grouped into three categories: 1) school districts in which strategies were completed, in progress, or being done by most schools; 2) those in which they were not being done by most schools, no action had been taken, or the response was not applicable; and 3) unknown or missing. Analyses were conducted using R (version 1.4.1106; R Foundation). This activity was reviewed by CDC and conducted consistent with applicable federal law and CDC policy.^††^

A total of 8,410 school districts (64.2%) representing an estimated 61.7% of enrolled public school students responded to ventilation-related questions (Table 1). Among responding school districts and national K–12 public school districts,^§§^ most (53%) were classified as rural, and the largest percentage (37%) were in the Midwest region. Responding school districts were similar to all U.S. K–12 public school districts in terms of U.S. Census Bureau region, locale, and poverty level distributions. Among the four ventilation improvements examined, maintaining continuous airflow in classrooms was reported by just over one half (50.7%) of school districts; one third (33.9%) reported having HVAC system improvements in progress or completed, more than one quarter (28.0%) reported planned or completed use of HEPA-filtered in-room air cleaners, and 8.2% reported UVGI improvements planned or completed in most schools (Table 2). The use of HEPA-filtered in-room air cleaners was also more frequently reported by districts in cities (33.1%) than in those in suburban (29.9%), town (27.4%), and rural (26.9%) districts. UVGI improvements were reported less frequently overall, and by 8.9% of rural school districts, followed by 8.4% of city, 8.0% of town, and 6.6% of suburban districts.

**TABLE 1:** Number and percentage of K–12 public school districts surveyed* about COVID-19 prevention strategies (N = 8,410), by region, locale, and poverty level compared with total national K–12 public school districts — United States, August–December 2022

Characteristic	No. (%)			
	Participating districts	Estimated students represented by participating districts	National districts^†^	Estimated students represented by national districts^§^
**Total**	**8,410**	**30,656,251**	**13,103**	**49,670,152**
**Region^¶^**				
Northeast	1,499 (17.8)	3,850,328 (12.6)	2,703 (20.6)	6,523,546 (13.1)
Midwest	3,137 (37.3)	6,590,076 (21.5)	4,728 (36.1)	10,349,402 (20.8)
South	2,033 (24.2)	10,747,723 (35.1)	3,082 (23.5)	20,015,472 (40.3)
West	1,741 (20.7)	9,468,124 (30.9)	2,589 (19.8)	12,482,171 (25.1)
Locale**				
City	487 (5.8)	9,373,912 (30.6)	747 (5.7)	14,916,562 (30.0)
Rural	4,482 (53.3)	4,962,213 (16.2)	7,009 (53.5)	7,852,801 (15.8)
Suburb	1,892 (22.5)	12,633,748 (41.2)	3,013 (23.0)	21,409,409 (43.1)
Town	1,549 (18.4)	3,686,378 (12.0)	2,333 (17.8)	5,491,380 (11.1)
**Poverty level^††^**				
Low	2,643 (31.4)	10,936,819 (35.7)	4,367 (33.4)	16,817,905 (33.9)
Mid	2,824 (33.6)	10,465,957 (34.1)	4,366 (33.3)	16,760,744 (33.7)
High	2,941 (35.0)	9,253,450 (30.2)	4,366 (33.3)	16,091,478 (32.4)

**Abbreviations:** NCES = National Center for Education Statistics; SAIPE = Small Area Income Poverty Estimates.

* MCH Strategic Data Survey. https://www.mchdata.com/

^†^ National districts are public school districts for which 2021 U.S. Census Bureau SAIPE data (https://www.census.gov/data/datasets/2021/demo/saipe/2021-school-districts.html) and NCES locale data (https://nces.ed.gov/programs/edge/Geographic/LocaleBoundaries) were available.

^§^ Enrollment data were not available for 191 districts.

^¶^ Puerto Rico does not have an assigned U.S. Census Bureau region; its one public school district has an estimated 0.6% of students (n = 299,561) represented by national districts.

** Locale was based on NCES locale classification.

^††^ Four districts did not have students aged 5–17 years in SAIPE data and were not assigned a poverty level; two were participating districts for which MCH Strategic Data estimated combined enrollment of 25 students.

**TABLE 2:** Strategies to improve ventilation in U.S. K–12 public school districts (N = 8,410), by locale,* U.S. Census Bureau region,^†^ and poverty level — United States, August–December 2022^§^

Ventilation intervention^¶^ and improvement status	No. (%)											
	NCES locale*				U.S. Census Bureau region^†^				School district poverty level**			Total
	City	Rural	Suburb	Town	Northeast	Midwest	South	West	Low	Mid	High	
**Replaced or upgraded HVAC systems**												
Completed or in progress by most schools	189 (38.8)	1,517 (33.8)	663 (35.0)	483 (31.2)	491 (32.8)	1,012 (32.3)	653 (32.1)	696 (40.0)	879 (33.3)	939 (33.3)	1,034 (35.1)	**2,852 (33.9)**
No action taken/Not applicable	63 (12.9)	1,269 (28.3)	254 (13.4)	325 (21.0)	209 (13.9)	876 (27.9)	454 (22.3)	372 (21.4)	560 (21.1)	698 (24.7)	652 (22.2)	**1,911 (22.7)**
Unknown	235 (48.3)	1696 (37.8)	975 (51.5)	741 (47.8)	799 (53.3)	1,249 (39.8)	926 (45.5)	673 (38.6)	1,204 (45.6)	1,187 (42.0)	1,255 (42.7)	**3,647 (43.4)**
**Installed or used HEPA filtration systems in classrooms or student dining areas**												
Completed or in progress by most schools	161 (33.1)	1,207 (26.9)	566 (29.9)	424 (27.4)	401 (26.8)	713 (22.7)	520 (25.6)	724 (41.6)	719 (27.2)	759 (26.9)	879 (29.9)	**2,358 (28.0)**
No action taken/Not applicable	71 (14.6)	1,455 (32.5)	261 (13.8)	348 (22.5)	226 (15.1)	1,031 (32.9)	518 (25.5)	360 (20.7)	655 (24.8)	761 (26.9)	718 (24.4)	**2,135 (25.4)**
Unknown	255 (52.4)	1,820 (40.6)	1,065 (56.0)	777 (50.2)	872 (58.2)	1,393 (44.4)	995 (48.9)	657 (37.7)	1,269 (48.0)	1,304 (46.2)	1,344 (45.7)	**3,917 (46.6)**
**Installed UVGI in high-risk and student dining areas, or where options for ventilation are limited**												
Completed or in progress by most schools	41 (8.4)	398 (8.9)	124 (6.6)	124 (8.0)	92 (6.1)	222 (7.1)	215 (10.6)	158 (9.1)	183 (6.9)	191 (6.8)	313 (10.6)	**687 (8.2)**
No action taken/Not applicable	153 (31.4)	2,428 (54.2)	706 (37.3)	715 (46.2)	582 (38.8)	1,686 (53.7)	902 (44.4)	832 (47.8)	1,246 (47.1)	1,427 (50.5)	1,328 (45.2)	**4,002 (47.6)**
Unknown	293 (60.2)	1,656 (36.9)	1,062 (56.1)	710 (45.8)	825 (55.0)	1,229 (39.2)	916 (45.0)	751 (43.1)	1,214 (45.9)	1,206 (42.7)	1,300 (44.1)	**3,721 (44.2)**
**Maintained continuous movement of air supply/airflow through the building during active hours^††^**												
Being done by most schools	123 (49.8)	1,195 (52.6)	516 (50.9)	370 (45.5)	429 (53.6)	747 (46.5)	463 (46.1)	565 (60.2)	688 (51.3)	706 (48.9)	810 (51.8)	**2,204 (50.7)**
No action taken/Not being done by most schools	30 (12.1)	450 (19.8)	99 (9.8)	123 (15.1)	59 (7.4)	343 (21.4)	186 (18.5)	114 (12.2)	206 (15.4)	265 (18.4)	231 (14.8)	**702 (16.1)**
Unknown	94 (38.1)	629 (27.7)	398 (39.3)	321 (39.4)	312 (39.0)	515 (32.1)	356 (35.4)	259 (27.6)	447 (33.3)	472 (32.7)	522 (33.4)	**1,442 (33.1)**

**Abbreviations**: HEPA = high-efficiency particulate air; HVAC = heating, ventilation, and air conditioning; NCES = National Center for Education Statistics; SAIPE = Small Area Income Poverty Estimates; UVGI = ultraviolet germicidal irradiation.

* https://nces.ed.gov/programs/edge/Geographic/LocaleBoundaries

^†^ https://www.census.gov/programs-surveys/economic-census/guidance-geographies/levels.html#par_textimage_34

^§^ https://www.mchdata.com/

^¶^ Respondents were asked, “Since the start of the COVID-19 pandemic, which of the following steps to increase ventilation or filter/clean air apply to most schools (at least 50%) in your district?”

** Two districts were not assigned poverty tertiles because they did not include any children or adolescents aged 5–17 years, according to SAIPE school district estimates for 2021.

^††^ Included opening windows and doors, using fans, and adjusting thermostats or central controls. Only responses on and after October 26, 2022, were included for analysis, which reduced the number of public school districts surveyed to 4,348.

By U.S. Census Bureau region, districts in the West more frequently reported continuous airflow (60.2%), HVAC (40.0%), and in-room air cleaners with HEPA filters (41.6%) ventilation improvements in most schools than did those in the Northeast (53.6%, 32.8%, and 26.8%, respectively), Midwest (46.5%, 32.3%, and 22.7%, respectively), and South (46.1%, 32.1%, and 25.6%, respectively). UVGI improvements were more commonly reported by school districts in the South (10.6%) than by those in West (9.1%), Midwest (7.1%), and Northeast (6.1%) regions. High-poverty school districts more frequently reported each of the four ventilation improvements in most schools than did low- and mid-poverty school districts, with 51.8% reporting maintaining continuous airflow, 35.1% reporting HVAC, 29.9% reporting HEPA-filtered in-room air cleaners, and 10.6% reporting UVGI improvements. For all possible interventions, unknown or missing responses accounted for 28%–60% of responses.

## Discussion

This report highlights four strategies public school districts have used to reduce transmission of respiratory infections through improved ventilation and facilitate safe in-school learning (*4*), as well as differences in strategy used by U.S. Census Bureau region, NCES locale, and school district poverty level. The most frequently reported ventilation strategy, maintaining continuous movement of airflow in school buildings, was also the least expensive to implement and was reported by approximately one half of school districts. More costly strategies such as installation and use of in-room air cleaners with HEPA filters and installation of UVGI were less frequently reported. That none of the four ventilation strategies was reported by more than approximately one half of school districts underscores the ongoing opportunity to improve indoor air quality among K–12 school buildings in the United States. CDC guidance for COVID-19 prevention to support safe in-person learning and improving ventilation in buildings, and the Environmental Protection Agency’s Indoor Air Quality Guide for Schools kit highlight various ways to improve ventilation, such as regular air filter replacement and moving barriers that might interfere with airflow (*1*,*5*,*6*).

Rural school districts less frequently reported replacing or upgrading HVAC systems and using HEPA-filtered in-room air cleaners than did school districts in other locales. This difference might be due to limitations in resource availability and difficulty finding contractors available and willing to complete capital improvements (*7*). In addition, rural schools might be more likely to use natural ventilation, such as opening windows, than are suburban and city schools because of less exposure to noise and air pollution in rural areas or simply having windows that can be opened. This finding is supported by an early 2022 report which found that lower-cost strategies were more frequently reported by schools overall, with rural schools least likely to report implementing resource-intensive ventilation strategies (*8*). School districts in the West region were more likely to report replacing or upgrading HVAC systems than were those in the Northeast, Midwest, and South regions, possibly because the buildings were newer and more amenable to implementation of technological improvements compared with older buildings in other regions. High-poverty school districts more frequently implemented all ventilation strategies compared with mid- and low-poverty school districts; these districts might have been prioritized and might have more experience applying for federal funding. A recent report found that high-poverty schools were more likely to use federal funds to undertake ventilation improvements (*8*).

The findings in this report are subject to at least six limitations. First, because ventilation strategies were reported by school district administrative staff members, responses might be influenced by respondents’ level of awareness of ventilation strategies used within their district. Limited awareness might be reflected by the high percentage of unknown responses to survey questions, resulting in a likely underreporting of ventilation improvements implemented. Second, these data were captured at the school district level. School-level variation in implementation of ventilation strategies within school districts might exist, but was not able to be examined. Third, strategies examined were not exhaustive, and school districts might have implemented additional improvements that were not identified by this survey. Fourth, although this study used a census-based approach to survey all U.S. K–12 public school districts, systematic differences between participating and nonparticipating schools could have affected the representativeness of these data; however, the distribution of participating school districts by U.S. Census Bureau region, NCES locale, and poverty level was similar to that of all U.S. K–12 public school districts. Fifth, without knowledge of the baseline ventilation status of participating school districts, a complete assessment of ventilation improvements was not possible. Finally, energy consumption before and during the COVID-19 pandemic was not evaluated as a means to measure the expense of adjusting thermostats and central controls to school districts.

Substantial federal funding remains available for ventilation improvements in school buildings that have been shown to reduce SARS-CoV-2 transmission^¶¶^ (*4*). Such improvements are part of a multicomponent approach to enhancing the school environment and could have benefits for COVID-19 and other airborne infectious disease prevention. Ventilation improvements can also reduce asthma exacerbations and allergy symptoms and have been linked to better academic outcomes for students (*9*); such improvements might also protect schools from extreme weather events, which have been shown to result in approximately one third of unplanned school closures that result in transition to remote learning (*10*), highlighting a need for adequate heating and cooling in school buildings as seasons change. Ventilation improvements can improve infectious and noninfectious disease outcomes for students and staff members (*4**,**10*).

Combined with staying current with COVID-19 vaccinations, staying home when sick, practicing proper hand hygiene and respiratory etiquette (including masking when appropriate), ventilation is part of a comprehensive approach to reducing COVID-19 spread and maintaining safe, in-person learning. Public health and education professionals can support districts in undertaking ventilation improvements now that might lead to far-reaching improvements among a variety of student and staff member health outcomes. Ensuring equitable access to resources, support, and other facilitators of ventilation improvements is important given identified geographic and socioeconomic disparities.

**Summary**

**What is already known about this topic?**

To reduce school transmission of SARS-CoV-2, K–12 public school districts implemented ventilation improvements (replacing or upgrading ventilation systems, installing filtration systems, installing ultraviolet germicidal irradiation devices, or improving airflow). Federal funding remains available for ventilation upgrades.

**What is added by this report?**

None of the ventilation strategies examined was reported by >51% of school districts. Implementation of ventilation improvements varied by school district U.S. Census Bureau region, geographic locale, and poverty level. High-poverty school districts reported implementation of the highest percentage of strategies.

**What are the implications for public health practice?**

Many public school districts have not taken steps to improve school building ventilation. Equitable access and support might be needed to assist school districts in their efforts to prevent respiratory infections through ventilation improvements.

## Footnotes

* The continuous airflow question was amended after the survey was launched. Only responses from October 26, 2022, onward were included in the analysis.

† Surveyed school districts included local education agency district types 1 (regular local school district not part of supervisory union), 2 (regular local school district part of supervisory union), and 7 (independent charter district). https://nces.ed.gov/ccd/commonfiles/glossary.asp

§
https://www.census.gov/programs-surveys/economic-census/guidance-geographies/levels.html#par_textimage_34

¶ NCES locales are divided into four types: rural, town, suburban, and city. https://nces.ed.gov/programs/edge/Geographic/LocaleBoundaries

**
https://www.census.gov/data/datasets/2021/demo/saipe/2021-school-districts.html

†† 5 C.F.R. part 46.102(l)(2), 21 C.F.R. part 56; 42 U.S.C. Sect. 241(d); 5 U.S.C. Sect. 552a; 44 U.S.C. Sect. 3501 et seq.

§§ National districts are public school districts for which 2021 U.S. Census Bureau SAIPE data (https://www.census.gov/data/datasets/2021/demo/saipe/2021-school-districts.html) and NCES locale data (https://nces.ed.gov/programs/edge/Geographic/LocaleBoundaries) were available.

¶¶
https://oese.ed.gov/offices/american-rescue-plan/american-rescue-plan-elementary-and-secondary-school-emergency-relief
